# Large Scale Solid Phase Synthesis of Peptide Drugs: Use of Commercial Anion Exchange Resin as Quenching Agent for Removal of Iodine during Disulphide Bond Formation

**DOI:** 10.1155/2012/323907

**Published:** 2012-10-15

**Authors:** K. M. Bhaskara Reddy, Y. Bharathi Kumari, Dokka Mallikharjunasarma, Kamana Bulliraju, Vanjivaka Sreelatha, Kuppanna Ananda

**Affiliations:** ^1^Chemical Research Division, Mylan Laboratories Ltd., Anrich Industrial Estate, Bollaram, Hyderabad 502325, India; ^2^Department of Chemistry, College of Engeenering, Jawaharlal Nehru Technological University Hyderabad, Kukatpally, Hyderabad 500085, India

## Abstract

The S-acetamidomethyl (Acm) or trityl (Trt) protecting groups are widely used in the chemical synthesis of peptides that contain one or more disulfide bonds. Treatment of peptides containing S-Acm protecting group with iodine results in simultaneous removal of the sulfhydryl protecting group and disulfide formation. However, the excess iodine needs to be quenched or adsorbed as quickly as possible after completion of the disulfide bond formation in order to minimize side reactions that are often associated with the iodination step. We report here a simple method for simultaneous quenching and removal of iodine and isolation of disulphide bridge peptides. The use of excess inexpensive anion exchange resin to the oxidized peptide from the aqueous acetic acid/methanol solution affords quantitative removal of iodine and other color impurities. This improves the resin life time of expensive chromatography media that is used in preparative HPLC column during the purification of peptide using preparative HPLC. Further, it is very useful for the conversion of TFA salt to acetate *in situ*. It was successfully applied commercially, to the large scale synthesis of various peptides including Desmopressin, Oxytocin, and Octreotide. This new approach offers significant advantages such as more simple utility, minimal side reactions, large scale synthesis of peptide drugs, and greater cost effectiveness.

## 1. Introduction

Many naturally occurring peptides contain intradisulphide bridges, which play an important role in biological activities. Disulfide bond-containing peptides have long presented a particular challenge for their chemical synthesis [1]. There are many ways to form a disulphide bridge in solution, and solid phase synthesis is well known and widely used in peptide community. Solution phase cyclization is most commonly carried out using air oxidation and or mild basic conditions [2]. The cyclization of free thiols by air oxygen usually leads to low yields of target peptides (10–15%) [3]. The application of potassium ferricyanide [4] or dimethyl sulphoxide [5] usually results in homogeneous reaction mixtures, and yields of cyclic peptides are considerably higher. However, a multistage purification of peptide is necessary for the removal of excess of these oxidative reagents [6]. Beginning with the initial discovery by Kamber et al. [7] that in peptides, where thiols are protected by Acm groups, iodine offered the potential to carry out removal and simultaneous oxidative disulphide bond formation in one-single step, several disulphide-bonded peptides have been synthesized using this strategy [8]. Unfortunately, side reactions are often associated with this reaction including, for example, iodination of some sensitive residues such as Tyr, Met, and Trp [9]. 

To help limit these, excess iodine should be quenched or adsorbed as quickly as possible after completion of the disulfide bond formation by addition of sodium bisulfite [9], sodium thiosulfate [10], ascorbic acid [11], powdered zinc dust, activated charcoal [12], or by dilution with water followed by extraction with carbon tetrachloride [13]. However, the quenching reagents themselves can sometimes cause additional side reactions including formation of thiosulfate adduct of the peptide [11]. The large volumes of highly diluted aqueous peptide solutions, post-Acm removal, can also encumber the subsequent RP-HPLC purification. Further, lyophilization of the peptide solution can result in concentration of unremoved iodine leading to additional side reactions. Thus, improved method for quenching of the iodine and subsequent isolation of disulphide peptides are desirable.

Ion exchange resins are insoluble polymers that contain acidic or basic functional groups and have the ability to exchange counter ions within aqueous solutions surrounding them. Anion exchange resins have positively charged functional groups, and there exchanges negatively charged ions. While the best-known usage of ion exchange resins is in water treatment, pharmaceutical applications were recognized in the early 1950's when amberlite IRC-50 was used in successful purification of streptomycin. Over the years, ion exchange resins have found use as pharmacologically active ingredients in drug formulations. However, the ion exchange resins have also been used in pharmaceutical manufacturing for the isolation and purification of drugs and catalysis of reactions [14, 15].

Anionexchange resins are widely used to remove acidic molecules from solution. They are used for scavenging excess reagents, and byproducts have been exploited in the solution phase synthesis of chemical libraries [16]. Ion exchange resins are synthetic polymeric materials that contain basic or acidic groups that are able to interact with ionizable molecules to create insoluble complexes. A typical strongly basic anion exchange resin will have quaternary ammonium groups attached to a styrene and divinylbenzene copolymer [17]. It has been reported by many workers that polyiodide ions, such as triiodide and pentaiodide, exhibit extremely high affinity for quaternary ammonium strong-base anionexchange resins in aqueous solutions [18]. Here we report, the utility of the indions 830-S [19] commercially available, strongly basic quaternary ammonium anion exchange resin [20] as quenching agent to remove excess of iodine and iodide during disulphide bridge formation. It is further used for the salt exchange (TFA salt to Acetate) simultaneously in a single step. 

## 2. Materials and Methods

All amino acid derivatives and Rink amide resin were purchased from GL Biochem china.Scavengers, coupling agents and cleavage reagents [N, N-diisopropylcarbodiimide (DIC), N-diisopropyl-ethylamine (DIEA), 1-Hydroxybenzotriazole (HOBt), triisopropylsilane (TIS), piperidine, trifluoroacetic acid (TFA)] were Fluka products. Oxyma and COMU were procured from Sigma-Aldrich. Indion 830-S resin was purchased from Ion Exchange India Pvt Limited. The solvents used were all commercial grade.

**Peptide Synthesis**

**Figure 1:**
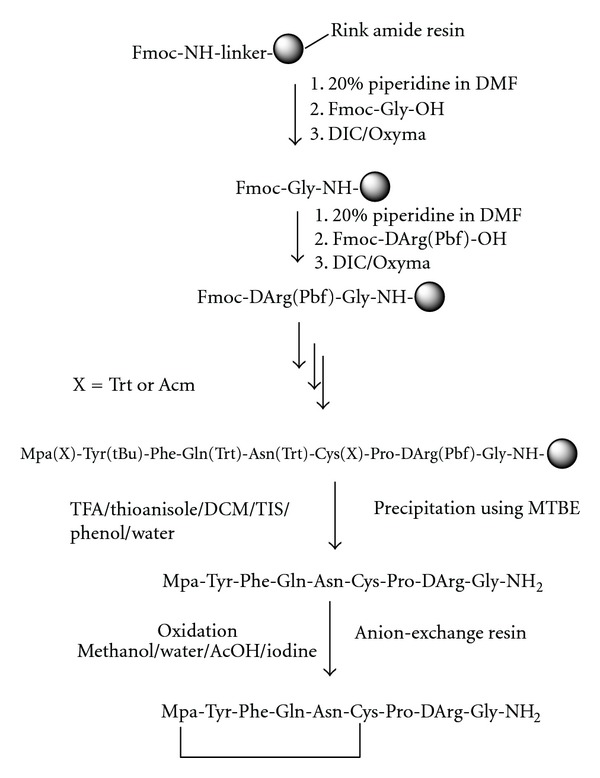
Scheme for solid phase synthesis of Desmopressin.

**Figure 2:**
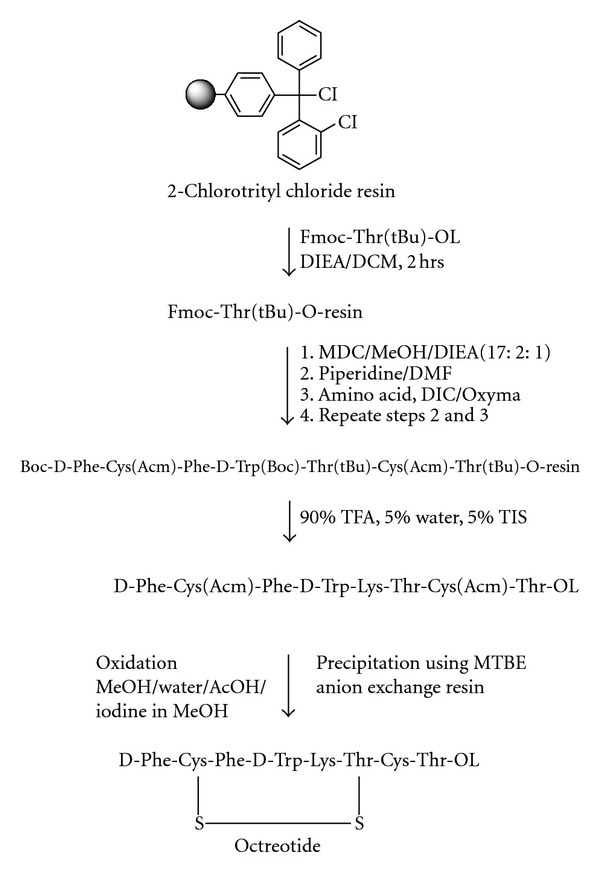
Solid phase synthesis of Octreotide.

**Figure 3:**
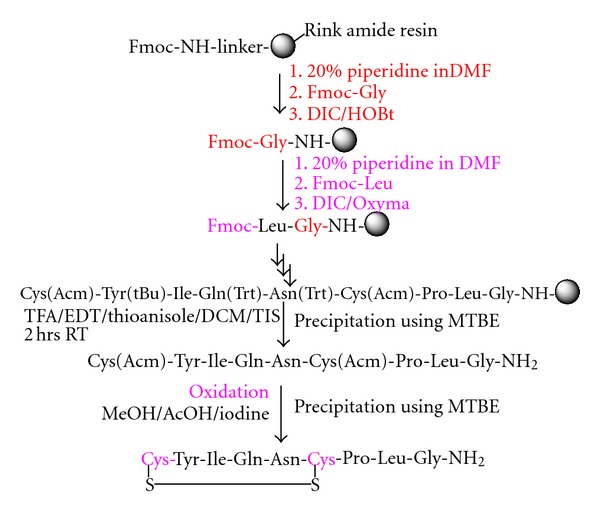
Solid phase synthesis of Oxytocin.

Peptides were synthesized manually by solid phase method by stepwise coupling of Fmoc-amino acids to the growing chain on a Rink amide resin (0.9–1.1 mmol/g, Figures 1 and 3 for Desmopressin and Oxytocin) and 2-chlorotrityl chloride resin (Figure 2, for Octreotide). The Fmoc-amino acids were used with the Trt and the Pbf as side chain protecting groups for Asn, Gln, Cys, and Arg, respectively. The couplings were done using DIC/HOBt, in a mixture of DMF/NMP (1 : 1), in most of the cases, the amino acids were coupled two fold excess. The completeness of each coupling reaction was monitored by Kaiser or Chloranil test. The individual coupling steps, if showing low coupling efficiency must be repeated prior to proceeding for deprotection and coupling with next amino acid of the sequence.

Octreotide was synthesized manually on 2-chlorotrityl chloride resin (substitution 0.90 mmol/g) by standard Fmoc solid phase synthesis strategy (Figure 2). Fmoc-Thr(tBu)-OL/Fmoc-Thr-OL was treated with the swelled 2-CTC resin in DCM in the presence of DIEA, and substitution level was determined by weight gain measurements and also by UV Method. It was found that Fmoc-Thr-OL gives better loading than Fmoc-Thr(tBu)-OL. This may be due to steric hindrance of t-butyl group. After the coupling of the first amino acid onto the resin, the unreacted linkers on the resin (polymer) are protected, to avoid the undesired peptide chain formation, with a solution of 5% DIEA and 10% methanol in DCM. This process of capping was performed after anchoring the first protected amino acid to the resin.

The complete synthesis was achieved by stepwise coupling of Fmoc-Amino acids to the growing peptide chain on the resin. All the couplings were carried out in DMF. The couplings were performed by dissolving the Fmoc-Amino acid (2 eq.) and HOBt (2 eq.) in DMF, and then DIC (2 eq.) was added. The reaction mixture was added to the resin and allowed to stir for 2 hrs. The efficiency of the coupling was monitored using the Kaiser Ninhydrin test. The coupling step was repeated if Kaiser test was found positive.

### 2.1. Cleavage of Peptide from Resin Along with Global Deprotection

The Octreotide resin (20 g) was swelled in DCM (50 mL) for 15 to 20 minutes under nitrogen at 25–30°C. The cocktail mixture (TFA (180 mL), water (10 mL), and TIS (10 mL)) was added to the resin and stirred for 2.5 hours at room temperature under nitrogen atmosphere. The reaction mixture was filtered and washed the resin with TFA (25 mL). The obtained filtrate was added into cold MTBE (500 ml, precooled to a temperature of 0–5°C) under stirring and allowed the temperature to rise more than 5°C. The reaction mixture was stirred for 45 minutes at 0–5°C. The obtained suspension was filtered, washed the solid with MTBE (500 mL), and dried the solid under nitrogen.

### 2.2. Purification of Mpa-Tyr-Phe-Gln-Asn-Cys-Pro-DArg-Gly-NH_2_

Peptide thiol (5 g) was slurried in a mixture of ethyl acetate as follows: ethanol (95 : 5) at 0°C for 1 hour. The reaction mixture was filtered and washed with ethyl acetate to afford 4.5 g of pure peptide thiol (~95%).

### 2.3. Conversion of Thiol to Disulphide

A 2% solution of iodine in methanol (12 mL) was added portion wise to a solution of the peptide free thiol (1 g) in 10% aq AcOH (10 mL), and the mixture was stirred at room temperature for 60 min. Reaction completion was confirmed by RP-HPLC. Anion exchange resin (10 g, Cl^−^ form) was added, and stirring was continued for 30 min. The suspension was then filtered to remove the resin, and the resin was washed further with additional small amounts of water. The combined filtrates were evaporated; the residue was suspended in water and lyophilized. The obtained products were analyzed by RP-HPLC and ESI-MS.

### 2.4. Disulphide Bridge Formation with Acm Protecting Groups

To a solution of semiprotected peptide in 80% aq methanol, was added slowly 10% solution of iodine in methanol till yellow color persists. The reaction is stirred at room temperature for 60 min. Reaction completion was confirmed by RP-HPLC. The excess of iodine was quenched with anion exchange resin, and stirring was continued for 30 min. The suspension was then filtered to remove the resin, and the resin was washed further with additional small amounts of water. The combined filtrates were evaporated; the residue was suspended in water and lyophilized. The obtained products were analyzed by RP-HPLC and ESI-MS.

### 2.5. Procedure for Conversion of Ion Exchange Resin to Acetate Form

Indion 830-S anion exchange resin (Chloride form, Cl^−^) was converted to acetate form by washing with IM aq NaOH, water, acetic acid, water, and methanol. The resin was dried and stored.

### 2.6. Procedure for Salt Exchange

The peptide in its trifluoroacetate form was dissolved in methanol water and added the anion exchange resin (acetate form) and stirred for 15 min. The resin is filtered and washed with methanol. The methanol is evaporated, and the product is lyophilized and monitored the acetate content by HPLC (Assay). The acetate content usually in the range of 4–6%.

### 2.7. Preparative HPLC Purification of Disulphide Bridge Peptides

The crude peptides were loaded on to preparative C18 column (50 × 250 mm, 100 A°). The peptide was purified on a preparative HPLC using gradient method by elution with a gradient comprising of buffer A: Water/Acetic acid (0.05%) and buffer B: Methanol/Acetic acid (0.05%). The peptides were eluted at around 30% of methanol, during the elution the fractions are collected at regular intervals. The collected fractions are assayed by HPLC to determine the purities, and fractions with desired purities are pooled together. The methanol was evaporated and the aqueous layer was lyophilized to obtain the peptides as white solid. The purified peptides were analyzed by RP-HPLC and mass determined by mass spectrometer. The purification achieved by this method utilizes the desired salt in a single purification step avoids the additional desalting step. Analytical HPLC was carried out on a 150 × 4.6 mm, 5 micron Purospher RP-18 column, flow rate 1 mL/min using a gradient conducted over 50 min. of 0.1% aqueous TFA and 0.1% TFA-CH_3_CN from 10–90%.

## 3. Results and Discussion 

Disulphide bridges play a crucial role in the folding and structural stabilization of many peptide and protein molecules, including many enzymes and hormones. The simplest approach to prepare disulphide-containing peptides involves complete deprotection and then careful oxidation to the properly folded product (Figure 1). The crude cleaved peptide generally needs to be treated with suitable reducing agents to ensure disulphide bridge is formed.

The resulting dithiols after cleavage of the peptide from resin was cyclized with a 0.1M I_2_ in methanol using a standard procedure. The reaction was monitored by RP-HPLC, and after completion of disulphide bond formation as evidenced by the disappearance of the starting peptide, the reaction is quenched with an anion exchange resin and stirred for 30 min's. The reaction turns colorless; the liberated iodide ions are adsorpted or exchanged to quaternary ammonium exchange resin [R-N^+^(CH_3_)_3_I^−^]. The resin is filtered and washed with methanol/water. The solvent is evaporated under reduced pressure, and the resulting residue is directly taken for preparative HPLC purification or dissolved in water and lyophilized. 

The peptides (crude) were isolated (Table 2) in ~80% yield by iodine oxidation followed by quenching with an anion exchange resin. The yield of peptides after preparative HPLC purification was 50–60% recovery of the peptides. There is no yield loss as confirmed by washing the used anion exchange resin with a mild base.

The ion exchange resin was washed thoroughly with water and methanol before use. The resin prewash step is necessary to ensure that no extractable material from resin contaminated the product when the later was mixed with the resin. Indion 830-S was stirred with water and methanol for 30 min's and filtered through a Buchner funnel. It was further washed with water and methanol and dried. The free thiol is added portion wise to a cooled mixture composed of (methanol : acetic acid : water) and stirred for 15 min at RT. Add iodine solution in methanol to the reaction slowly up to yellow color persists. The reaction is stirred for another 1 hr at room temperature and monitored by HPLC for disulphide bridge formation. No dimmer was detected as confirmed by HPLC analysis. After completion, the washed anion exchange resin was added into the iodine solution of the disulphide forming reaction mixture and stirred for 15–30 min's. The reaction mixture shows colorless suggesting quantitative removal of iodine and iodide ions [21]. It was filtered through Buchner funnel, and the solvent was evaporated (Residue). The residue was directly injected into preparative HPLC or by dissolving the residue with water and isolated the peptide by lyophilization. 

Iodine has proven its utility in forming disulphide bonds in Acm or trityl protected peptides. However, as soon as the disulphide bonds have been made, a safe practice is to quench the reactive iodine so as not to allow any side reaction on the peptide. One such common side reaction is the iodination of the phenolic side chain of tyrosine. Iodine can be quenched with thiosulphate or ascorbic acid, the quenched reaction mixture was lyophilized, and salts removed by gel filtration or RP-HPLC. However, caution must be exercised since unexpected side reactions have been observed following the use of quenching agents. 

Our initial attempts to use ascorbic acid as quenching agent lead to less pure peptides compared to the present method. The yields are low (Table 2), and the main issue is that during preparative HPLC purification, due to ascorbic acid, the separation was not good for higher loading of the peptide to preparative column.

In the present study, it has been established that an ion exchange resin was used to quench the excess of iodine used for disulphide bridge formation, there is no need to remove the salts by gel filtration, the reaction is neat and clean circumvents, the formation of impurities. Since an intramolecular disulphide must be made in conditions of high dilution, the postreaction handling of iodine and the cyclized peptides needs special consideration. A conventional gel filtration experiment is impractical. The resin quenching method is especially useful for the scale up of peptide drugs on a large scale. The method is utilized in the large scale manufacture of peptide drugs like desmopressin, Octreotide, and Oxytocin.

Cyclization was performed by additions of portions of the crude peptide to aqueous acetic acid methanol and stirred for 10 min's. 0.1M I_2_ in methanol was added slowly till a light yellow solution was obtained, and the peptide concentration was adjusted to 350 mL/g of the peptide with methanol. The cyclizations were tried with different concentrations and found that 350 mL/g was ideal as there was no dimmer/isomer formation. After the disulphide bridge was formed as monitored by HPLC. Anion exchange resin (chloride form) was added and stirring was continued for 30 min to remove iodide and excess of iodine. It was filtered through Buckner funnel, and the filtrate is evaporated. The residue is dissolved in water and lyophilized or directly taken for preparative HPLC purification. The purity of the peptide was assayed by HPLC and characterized by LCMS.

**Figure 4:**
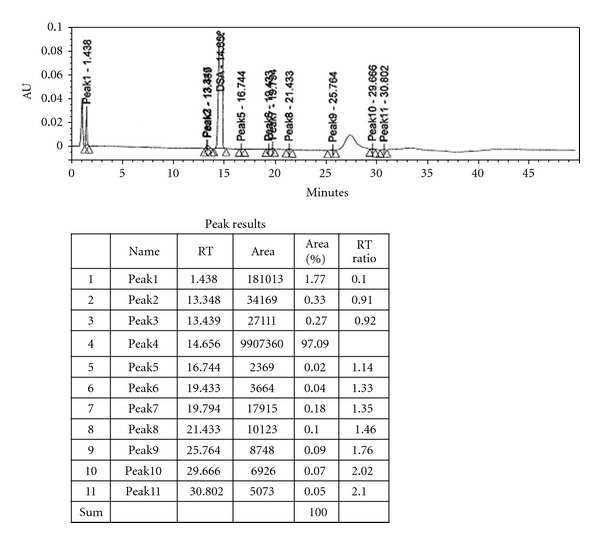
HPLC Chromatograph of desmopressin with incomplete removal of iodine In such cases, the peptide dissolved in water added the resin and stirred for 5 min's filtered and Lyophilized. The HPLC analysis (Figure 5) showed not even traces of iodine present.

**Figure 5:**
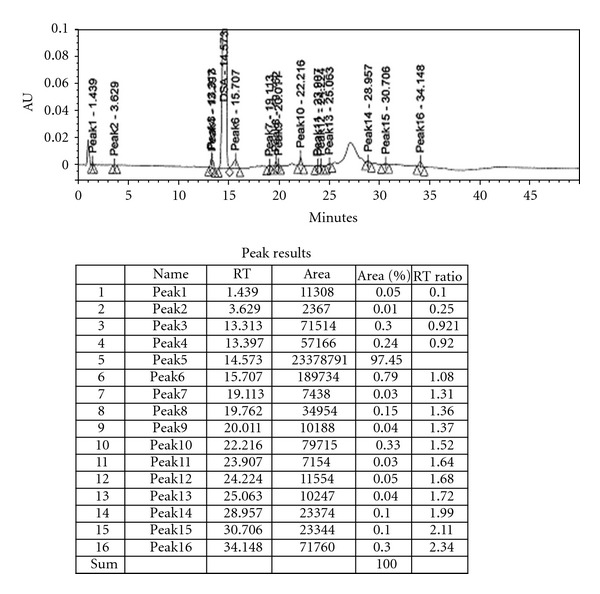
HPLC Chromatograph of desmopressin with complete removal of iodine.

The absence of iodine peak as monitored by HPLC (Figure 5) was found to be less than 0.5%. In an experiment that was carried out without the complete removal of iodine in the crude peptide, we found that even after purification using preparative HPLC the iodine peak was present (Figure 4).

Further, exchange resin is stirred with 0.1M iodine in methanol for 10 min's. The TLC analysis showed there are not even traces of iodine present; the resin beads were brown in color and completely adsorbed by the resin. 

The utility of other ion exchange resins, like Indion 860-S and Deuolite A-68, were tried with limited successes. It is found that a strongly, basic anion exchange resin is required for complete removal of iodine. Weak basic anion exchange resins were found to be less effective for the removal of iodine.

**Figure 6:**
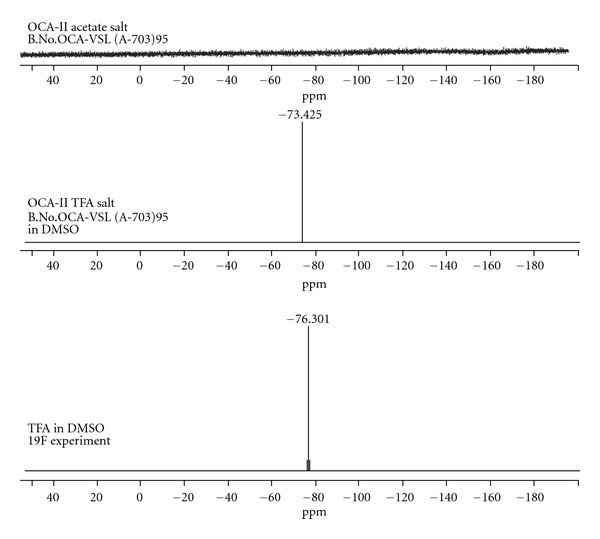
F NMR comparision of Octreotide acetate and Octreotide Trifluoro acetate.

The peptides synthesized on solid phase were cleaved using TFA leading to the formation of trifluoroacetate salt. Most of the peptide drugs in the market are having acetate salt. The salt exchange of trifluoroacetate to acetate was done using the anion exchange resin. The anion exchange resin after converting to its acetate form was added to peptide trifluoroactate salt dissolved in methanol/water and stirred for 30 mins and filtered, and the filtrate was lyophilized. The acetate was confirmed by HPLC (assay) and the IR for the absence of C-F stretching mode (1110–1200 cm^−1^). It is further confirmed by F NMR on the TFA peptide salt and compared the signal with an equivalent amount of TFA. The acetate peptide salt was at same concentration and compared the fluorine strength (Figure 6). Thus, the salt exchange is 100% as there is no signal corresponding to fluorine in acetate peptide.

The resin used in the process of the preparation of desmopressin acts as support material and is selected from Tentagel SRAM, 2-Chlorotrityl chloride resin (2-CTC), Rink amide resin (0.43 mmol/g), and Rink amide resin (1.1 mmol/g). The selection of polymeric support and attached linker is very critical for overall outcome of the solid phase peptide synthesis. The Rink amide resin (1.1 mmol/g) with amino methyl polystyrene type linker used in present application provides additional advantages over the other resins. Tentagel resin is also found to be very effective for the preparation of desmopressin and is comprising of grafted copolymers consisting of a low cross-linked polystyrene.

The advantage of using high load resin is that significantly more peptide for unit measure of beads could be produced with high load resins. This is a consequence of the fact that higher concentrations of reagents and reactants can be achieved with high load resins. Smaller vessel sizes could be employed to generate a given amount of peptide and at least 50% less wash solvents needed while using high loaded resins. For the scale up of the solid phase it is important to reduce the large amounts of reagents typically employed in solid phase peptide synthesis. 

The different resins (Table 1) were tried in desmopressin scale up optimization on large scale synthesis and found that Rink amide resin with 0.9–1.1 mmol/g was more useful for scale up in terms of yield and cost.

An ion exchange resin is a cost effective and used extensively for purifying water and food processing. Therefore, the use of ion exchange resin in the large scale peptide drug process is an advantageous. In comparison with the reported methods for the large scale synthesis of desmopressin, our method found to be advantageous in terms of scale up as well as isolation of peptide. The reported method utilizes two step purification process for crude peptide and a separate step for salt exchange. The present method describes the use of high loading resin for the synthesis, and anion exchange resin for iodine quenching makes the method more robust for the synthesis of peptide drugs. The yields as well as purities of the peptides were good when compared with other quenching agents. It offers a number of attractive advantages such as ease of application, minimal side reactions, and cost effectiveness.

## 4. Conclusion 

A new and simple method for simultaneous quenching and removal of excess of iodine and iodide ions from aqueous solution containing iodine and iodide ions and peptide isolation has been developed. The strong base anion exchange resin is more effective than the weak anion exchange resins. This simple method of quenching of iodine has been successfully applied to the synthesis of several peptides including Desmopressin, Oxytocin, and Octreotide. This method is very useful in large scale synthesis especially for disulphide bridge peptide API's to produce on a commercial scale. 

## Figures and Tables

**Table 1:** Resins of different loading capacity tried during Desmopressin scale up.

Resin	Particle size (*μ*m)	Matrix	Loading (mmol/g)
Tentagel SRAM	90	Poly(oxyethylene)-RAM Polymer bound	0.24
Rink amide resin	100–200	Amino methyl polystyrene crosslinked with 1% DVB	1.1
Rink amide resin	100–200	Amino methyl polystyrene crosslinked with 1% DVB	0.43
2-chlorotrityl chloride resin	100–200	Polystyrene crosslinked with 1% DVB	0.7–0.9

**Table 2:** Recovery yield of disulphide peptide quenched with anion exchange resin.

Peptide	Peptide conc (mL/g)	MeOH (%)	Crude purity (%)	Yield (%) crude	Yield (%) purified	Quenching agent
Desmopressin (Thiol)	350	85	90	80	50	Anion exchange resin
Desmopressin (Acm)	300	80	85	78	48	Anion exchange resin
Octreotide	200	95	88	85	58	Anion exchange resin
Oxytocin	450	80	82	82	47	Anion exchange resin
Desmopressin (Acm)	300	80	69	70 (including ascorbic acid)	30	Ascorbic acid
Octreotide	200	95	70	72 (including ascorbic acid)	32	Ascorbic acid

## Abbreviations

Acm: Acetimidomethyl
Trt: Trityl
TFA: Trifluoroacetic acid
Pbf: 2, 2, 4, 6, 7-pentamethyl-dihydrobenzofuran-5-sulfonyl
MTBE: Methyltert-butyl ether
CTC: Chlorotrityl chloride resin
Tyr: Tyrosine
Met: Methionine
Trp: Tryptophan.
